# Real-world clinical outcomes of CAR-T therapy from the Montefiore health system in the Bronx

**DOI:** 10.1007/s00277-025-06727-x

**Published:** 2025-11-13

**Authors:** Sonya Henry, Ahmed Abbasi, Aiman Hafeez, Aditi Vichare, R. Alejandro Sica, Tim Q. Duong

**Affiliations:** 1 Department of Radiology, Montefiore Health System and Albert Einstein College of Medicine, 1300 Morris Park Avenue, Bronx, NY 10461 USA; 2 Department of Oncology, Montefiore Health System and Albert Einstein College of Medicine, Bronx, NY USA

**Keywords:** Immunotherapy, Blood cancer, Neurological sequela, Cytokine release syndrome

## Abstract

Chimeric antigen receptor T-cell (CAR-T) therapy has transformed diffuse large B-cell lymphoma (DLBCL) treatment, but data in diverse urban populations remain limited. We report outcomes from a comprehensive cancer center in the Bronx. Montefiore Health System treated 79 DLBCL patients with CAR-T from March 2018 to July 2024. Demographics, comorbidities, socioeconomic factors, and acute complications, including immune effector cell neurotoxicity syndrome (ICANS), cytokine release syndrome (CRS), cardiotoxicity, and pulmonary events, were tabulated. Kaplan-Meier survival analysis was performed up to five years. Multivariate adjusted hazard ratios (aHR) were calculated for remission and 3-year mortality. CAR-T achieved complete remissions in 50.6%, partial remission in 16.5%, and progression in 22.8%. Cumulative mortality was 10.1% during treatment and 34.2% by five years. Complete remission reduced 3-year mortality (aHR 0.13; 95% CI 0.04–0.43). Complications included CRS (76.0%), ICANS (49.4%), neutropenic fever (58.2%), and sepsis (19.0%). ICANS correlated with CRS, ICU stay, and sepsis. Socioeconomic status was not linked to mortality, but comorbidities increased risk. CAR-T complete remission is an independent predictor of reduced mortality. High rates of complications, particularly CRS and ICAN, reinforce the need for vigilant monitoring and management strategies to enhance long-term outcomes.

**Supplementary Information:**

The online version contains supplementary material available at 10.1007/s00277-025-06727-x.

## Introduction

 Chimeric antigen receptor T-cell (CAR-T) therapy, since its FDA approval in 2017, has become a groundbreaking form of immunotherapy that has revolutionized the treatment of hematologic malignancies [1, 2]. This innovative approach involves genetically modifying a patient’s own T cells to express a synthetic receptor that specifically targets cancer cells. By harnessing the body’s immune system, CAR-T therapy offers a highly targeted and durable treatment option for patients with relapsed or refractory blood cancers, such as B-cell acute lymphoblastic leukemia (B-ALL), diffuse large B-cell lymphoma (DLBCL), and multiple myeloma [2]. The clinical success of this novel treatment, marked by high response rates and increased overall survival, has revolutionized cancer treatment paradigms, offering new hope when conventional therapies have failed.

However, alongside its remarkable efficacy, CAR-T therapy is associated with significant acute and long-term side effects [1, 2]. One of the most common and serious adverse effects is cytokine release syndrome (CRS) [3, 4], a systemic inflammatory response triggered by excessive immune activation following CAR-T cell infusion. CRS results from the widespread release of pro-inflammatory cytokines, such as interleukin-6, interleukin-1, interferon-gamma, and tumor necrosis factor-alpha. This immune dysregulation can lead to high fever, hypotension, hypoxia, and multi-organ dysfunction, with severe cases progressing to vascular leak syndrome and disseminated intravascular coagulation [5, 6]. These and other factors could lead to or associated with other side effects such as neurological (i.e., immune effector cell-associated neurotoxicity syndrome (ICANS)), cardiac, pulmonary, and gastrointestinal complications, as well as mortality [7–11]. Moreover, most studies to date have been from carefully selected clinical trials or studies. The long-term effects of CAR-T therapy and whether and how these complications contribute to long-term outcomes are poorly understood in real-world clinical settings, given the novelty of this therapy.

CAR-T therapy also incurs a substantial cost, which can restrict access for some population subgroups [12]. Prior research has highlighted disparities in health outcomes in hematologic malignancies among underserved populations [13]. One study evaluating the National Inpatient Sample database in 2018 concluded that recipients of CAR-T therapy are more likely to be White and residents of urban city centers [14]. Research on trends exploring the accessibility of CAR-T shows that higher-income households are more likely to receive CAR-T therapy [15]. Moreover, the higher prevalence of some major comorbidities, such as metabolic, renal, and cardiovascular diseases, among underserved population may contribute to poorer CAR-T treatment outcomes.

The goal of this study was to report the clinical outcomes of CAR-T therapy up to 5 years post-treatment in the Montefiore Health System in the Bronx, a NIH-designated comprehensive cancer center (real-world data). Our center serves a predominantly minority and lower-income population with high rate of medical comorbidities in the Bronx, which remains underrepresented in CAR-T literature. We analyzed the acute complications associated with CAR-T therapy, the rate of complete remission, all-cause mortality post-treatment, and identified risks (including socioeconomic status) for outcomes up to 5 years post-treatment.

## Methods

This study was approved by the Einstein-Montefiore Institutional Review Board with an exemption for informed consent and a HIPAA waiver (2024–16474). Data originated from the Montefiore Health System, which consists of multiple hospitals and outpatient clinics located in the Bronx and its environs, serving a highly diverse urban population. The Bronx is one of the socioeconomically disadvantaged counties in the United States. Data was extracted from the electronic medical record (EMR) via OMOP data model [16–27] as well as by chart review. The study period was from 03/30/2018 to 7/26/2024. This study focused on the use of CAR-T therapy (Axicabtagene Ciloleucel), predominantly on diffuse large B-cell lymphoma (DLBCL).

### Variables

Demographic data (age, sex, ethnicity, race), cancer types, prior treatments and outcomes (such as prior chemotherapy), pre-existing comorbidities (obesity, smoking, hypertension, chronic kidney disease (CKD), type 2 diabetes mellitus (T2DM), chronic kidney disease (CKD), congestive heart failure (CHF), myocardial infarct, respiratory failure, chronic obstructive pulmonary disease (COPD), and asthma by ICD-10 codes) were tabulated. Quartile median incomes, insurance status (private, Medicare, Medicaid, Other), and unmet social needs were also tabulated.

Acute CAR-T treatment complications (cytokine release syndrome (CRS), neurological (ICAN), cardiac, pulmonary, and gastrointestinal (GI)) complications based on clinical notes were also tabulated. Intensive care unit admission (ICU), sepsis, and neutropenic fever) were also recorded.

Laboratory data were extracted for patients at pre (0–3 months prior CAR-T and 3–6 months prior) and during (0–3 months post CAR-T). Values within each time window were averaged for each patient to generate a single pre- and post-treatment value. Labs included complete blood count and differential (e.g., white blood cell count, hemoglobin, hematocrit, lymphocytes, monocytes, neutrophils, and platelets), coagulation tests (prothrombin time, activated partial thromboplastin time, and international normalized ratio (INR)), kidney function markers (blood urea nitrogen (BUN) and creatinine (Cr)), liver function tests (aspartate aminotransferase (AST), alanine aminotransferase (ALT), and lactate dehydrogenase (LDH), total bilirubin, and albumin), electrolytes (magnesium, calcium, phosphate, and sodium), and the inflammatory marker C-reactive protein (CRP).

Blood marker troponin-I (TNT-I) and ejection fraction (EF from echocardiogram) were measured at pre (0–3 months prior CAR-T and 3–6 months prior) and during (0–3 months post CAR-T) CAR-T therapy. TNT-T, which is less specific to acute cardiac injury, was rarely measured and thus not analyzed.

### Outcomes

Primary outcomes were complete remission (CR) and all-cause mortality at 3 years post-CAR-T treatment. Kaplan-Meier survival analysis for mortality up to 5 years post-CAR-T treatment was also performed. For CR, univariate odds ratios were first obtained to reduce data dimensionality, and only variables with *p* < 0.05 in the univariate analysis were included in the multivariate model to calculate adjusted odds ratios (aOR) with 95% confidence intervals (CI). For mortality, adjusted hazard ratios (aHR) were estimated using multivariate Cox proportional hazards models, following the same data dimensionality approach. The all-cause mortality hazards model included CAR-T CR status as a covariate. Note that results from *prior* CAR-T treatment were not included in the multivariate analysis because most patients had progression of disease (POD) or relapse prior to CAR-T, for which CAR-T therapy was given.

Secondary outcomes included acute CAR-T treatment complications stated above. Correlation analyses of neurological (ICANS), cardiac, pulmonary, and GI complications with ICU, sepsis, neutropenic fever, and CRS were performed.

### Statistical analysis

Statistical analyses were conducted using Python libraries such as Statsmodels and Lifelines, as well as the survival package in R. Categorical variables representing patient demographic characteristics and comorbidities were compared using Pearson χ² or Fisher’s exact tests. Two tailed t-tests were utilized for continuous variables. The Wilcoxon signed-rank test was used for non-parametric paired comparisons of laboratory values in patients with both pre- and post-treatment measurements. Patients with missing laboratory values were excluded from those analyses.

Univariate and multivariate regression analyses were performed for both CR and mortality outcomes. Variable selection for multivariate models was based on univariate significance (*p* < 0.05) and correlation and VIF-based exclusion of collinear variables.

Laboratory values were dichotomized based on standard clinical reference ranges for inclusion in regression models. Values were classified as “high” if they exceeded the upper limit of normal and “low” if they fell below the lower limit using cutoffs defined in Supplemental Table 1. Dichotomized laboratory variables were included as covariates in univariate and multivariate analyses.

For mortality, the proportional hazards assumption was first tested and confirmed for multivariate Cox proportional hazard validity using Schoenfeld residuals. A p-value < 0.05 was considered statistically significant unless otherwise specified.

## Results

Table 1 shows the characteristics of 79 CAR-T patients. The mean age was 60.86 ± 14.22 years, with 54% male. Essentially, all patients had DLBCL. Prior to CAR-T, 41.77% of patients showed disease progression, and 40.51% had relapsed, 15% had at least 2 chemo lines, 35% had mutations, and 23.75% had prior autologous stem cell transplant. The top five comorbidities were hypertension, diabetes, smoking, obesity, and CHF.

**Table 1:** Patient profiles of all patients treated with CAR-T (*n* = 79)

Age Mean (SD)	60.86 (14.22)
Male, No (%)	43 (54.43%)
Non-Hispanic Black	19 (24.05%)
Non-Hispanic White	26 (32.91%)
Hispanic	23 (29.11%)
Other race/Unknown	11 (13.92%)
Type of cancers	
DLBCL	62 (78.48%)
Transformed to DLBCL	10 (12.66%)
DLBCL + Other	3 (3.8%)
Other	4 (5.06%)
Mutation (Double hit or more)	27 (34.18%)
Prior > 2 chemo lines	12 (15.19%)
Prior ASCT	19 (24.05%)
Prior treatment status	
Complete Remission	1 (1.27%)
Partial Remission	12 (15.19%)
Progression of Disease	33 (41.77%)
Relapse	32 (40.51%)
Unknown	1 (1.27%)
Comorbidities	
Obesity	23 (29.11%)
Smoking	34 (43.04%)
Hypertension	48 (60.76%)
Diabetes2	25 (31.65%)
CKD	10 (12.66%)
CHF	18 (22.78%)
Myocardial infarct	5 (6.33%)
Respiratory Failure	16 (20.25%)
COPD	16 (20.25%)
Asthma	11 (13.92%)
Insurance	
Medicaid	28 (35.44%)
Medicare	26 (32.91%)
Private	21 (26.58%)
Self-Pay	4 (5.06%)
Income	
Q1: $30,741 – $42,639	20 (25.32%)
Q2: $42,639 – $66,173	19 (24.05%)
Q3: $66,173 – $89,118	19 (24.05%)
Q4: $89,118 – $229,671	18 (22.78%)
Unknown Zip Code:	3 (3.8%)
Acute CAR-T treatment complications	
CRS	60 (75.95%)
Neutropenic Fever	46 (58.23%)
ICU	15 (18.99%)
Sepsis	15 (18.99%)
Neurological (ICANs) complications	39 (49.37%)
Cardiac Complications	8 (10.13%)
Pulmonary Complications	9 (11.39%)
GI Complications	8 (10.13%)
Response to CAR-T treatment	
Complete Remission	40 (50.63%)
Partial Remission/Response	13 (16.46%)
Progression of Disease	18 (22.78%)
Mortality Outcomes	
Died During Treatment	17 (21.52%)
1-year mortality	19 (24.05%)
2-year mortality	27 (34.18%)
5-year mortality	43 (54.43%)

Acute CAR-T treatment complications included CRS (75.95%), neutropenic fever (58.23%), sepsis (18.99%), ICU treatment (18.99%), ICANS (49.37%), cardiotoxicity (10.13%), pulmonary (11.25%), and GI (10%) complications. CAR-T treatment showed 50.63% complete remission, 16.46% partial remission, and 22.78% progression of disease. The cumulative mortality during treatment and at 1-, 2-, and 5-year post–CAR-T treatment was 10.13%, 21.52%,24.05%, and 34.18%, respectively.

Table 2 shows the characteristics of patients grouped by CR versus non-CR, and 3-year survivors versus non-survivors. Compared to the non-CR group, the CR group had a higher proportion of patients with complete remission and fewer with progression of disease (*p* < 0.001) by definition. No other characteristics significantly differed between CR and non-CR groups (*p* > 0.05). Compared to non-survivors, survivors had a lower proportion of males (*p* < 0.01), and lower rates of CHF (*p* < 0.01), CKD (*p* < 0.01), ICU admission (*p* < 0.001), sepsis (*p* < 0.05), and cardiac complications (*p* < 0.05). Survivors were also less likely to have had progression of disease following CAR-T (*p* < 0.05). No significant differences were observed for age, race, smoking status, hypertension, diabetes, insurance status, and income between survivor and non-survivor groups (*p* > 0.05).

**Table 2:** Patient profiles for CR vs. non-CR, survivors and non-survivors, 3 years post CAR-T. Patients who died during treatments were not included. P values indicated by * <0.05, ** <0.01, *** <0.001

	CR (*N* = 40)	Non-CR (*N* = 31)	Survivors @ 3 yrs (*N* = 52)	Non-survivors @ 3yrs (*N* = 16)
Age > 60	23 (57.50%)	18 (58.06%)	29 (55.77%)	10 (62.50%)
Age = < 60	17 (42.50%)	13 (41.94%)	23 (44.23%)	6 (37.50%)
Male	14 (35.00%)	17.0 (54.84%)	17 (32.69%)	12.0 (75.00%)**
Black	6 (15.00%)	11.0 (35.48%)	12 (23.08%)	5.0 (31.25%)
White	16 (40.00%)	8.0 (25.81%)	19 (36.54%)	3.0 (18.75%)
Hispanic	12 (30.00%)	7.0 (22.58%)	12 (23.08%)	6.0 (37.50%)
Other race/Unknown	6 (15.00%)	5.0 (16.13%)	9 (17.31%)	2.0 (12.50%)
Type of cancers and treatment				
DLBCL	30 (75.00%)	25 (80.65%)	39 (75.00%)	14 (87.50%)
Transformed to DLBCL	5 (12.50%)	4 (12.90%)	8 (15.38%)	0 (0.00%)
DLBCL + Other	0 (0.00%)	0 (0.00%)	0 (0.00%)	0 (0.00%)
Other	4 (10.00%)	1 (3.23%)	4 (7.69%)	1 (6.25%)
Mutation (Double hit/more)	14 (35.00%)	12 (38.71%)	16 (30.77%)	9.0 (56.25%)
Prior > 2 Chemo Lines	7 (17.50%)	5 (16.13%)	10 (19.23%)	2.0 (12.50%)
Prior ASCT	12 (30.00%)	5 (16.13%)	13 (25.00%)	3.0 (18.75%)
Prior Treatment Status				
Complete Remission	0 (0.00%)	1 (3.23%)	0 (0.00%)	1 (6.25%)
Partial Remission	9 (22.50%)	3 (9.68%)	7 (13.46%)	5 (31.25%)
Progression of Disease	12 (30.00%)	16 (51.61%)	19 (36.54%)	7 (43.75%)
Relapse	19 (47.50%)	10 (32.26%)	25 (48.08%)	3 (18.75%)*
Unknown	0 (0.00%)	1 (3.23%)	1 (1.92%)	0 (0.00%)
Pre-existing comorbidities				
Obesity	14 (35.00%)	7.0 (22.58%)	11 (21.15%)	9.0 (56.25%)*
Smoking	21 (52.50%)	9.0 (29.03%)	22 (42.31%)	7.0 (43.75%)
Hypertension	27 (67.50%)	16.0 (51.61%)	30 (57.69%)	11.0 (68.75%)
Diabetes2	13 (32.50%)	9.0 (29.03%)	14 (26.92%)	8.0 (50.00%)
CKD	3 (7.50%)	4.0 (12.90%)	2 (3.85%)	5.0 (31.25%)**
CHF	7 (17.50%)	7.0 (22.58%)	5 (9.62%)	8.0 (50.00%)**
Myocardial infarct	2 (5.00%)	3.0 (9.68%)	1 (1.92%)	4.0 (25.00%)**
Respiratory Failure	6 (15.00%)	5.0 (16.13%)	5 (9.62%)	5.0 (31.25%)*
Insurance				
Medicaid	15 (37.50%)	11 (35.48%)	18 (34.62%)	6 (37.50%)
Medicare	11 (27.50%)	10 (32.26%)	16 (30.77%)	4 (25.00%)
Private	10 (25.00%)	10 (32.26%)	16 (30.77%)	4 (25.00%)
Self Pay	3 (7.50%)	0 (0.00%)	1 (1.92%)	2 (12.50%)
Income				
Q1: $30,741 – $42,639	10 (25.00%)	7 (22.58%)	13 (25.00%)	4 (25.00%)
Q2: $42,639 – $66,173	10 (25.00%)	7 (22.58%)	10 (19.23%)	6 (37.50%)
Q3: $66,173 – $89,118	11 (27.50%)	7 (22.58%)	12 (23.08%)	4 (25.00%)
Q4: $89,118 – $229,671	8 (20.00%)	8 (25.81%)	14 (26.92%)	2 (12.50%)
Unknown Zip Code	1 (2.50%)	2 (6.45%)	3 (5.77%)	0 (0.00%)
Response to CAR-T treatment				
Complete Remission	-	-	29 (55.77%)	8 (50.00%)
Partial Remission/Response	0 (0.00%)	13 (41.94%)***	11 (21.15%)	2 (12.50%)
Progression of Disease	0 (0.00%)	18 (58.06%)***	12 (23.08%)	6 (37.50%)
Acute CAR-T treatment complications				
CRS	29 (72.50%)	25.0 (80.65%)	40 (76.92%)	12.0 (75.00%)
Neutropenic Fever	23 (57.50%)	15.0 (48.39%)	24 (46.15%)	12.0 (75.00%)*
ICU	7 (17.50%)	3.0 (9.68%)	9 (17.31%)	9.0 (16.98%)
Sepsis	4 (10.00%)	4.0 (12.90%)	6 (11.54%)	2.0 (12.50%)
Neurological (ICANs) complications	19 (47.50%)	12.0 (38.71%)	22 (42.31%)	8.0 (50.00%)
Cardiac Complications	3 (7.50%)	4.0 (12.90%)	3 (5.77%)	4.0 (7.55%)*
Pulmonary Complications	3 (7.50%)	3.0 (9.68%)	4 (7.69%)	2.0 (12.50%)
GI Complications	5 (12.50%)	2.0 (6.45%)	6 (11.54%)	1.0 (6.25%)
Mortality Outcomes				
1-year Mortality	2 (5.00%)	7 (22.58%)	0 (0.00%)	9 (56.25%)
2-year Mortality	4 (10.00%)	7 (22.58%)	0 (0.00%)	11 (68.75%)
3-year Mortality	8 (20.00%)	8.0 (25.81%)	0 (0.00%)	16.0 (100%)

### Mortality and CR

**Fig. 1:**
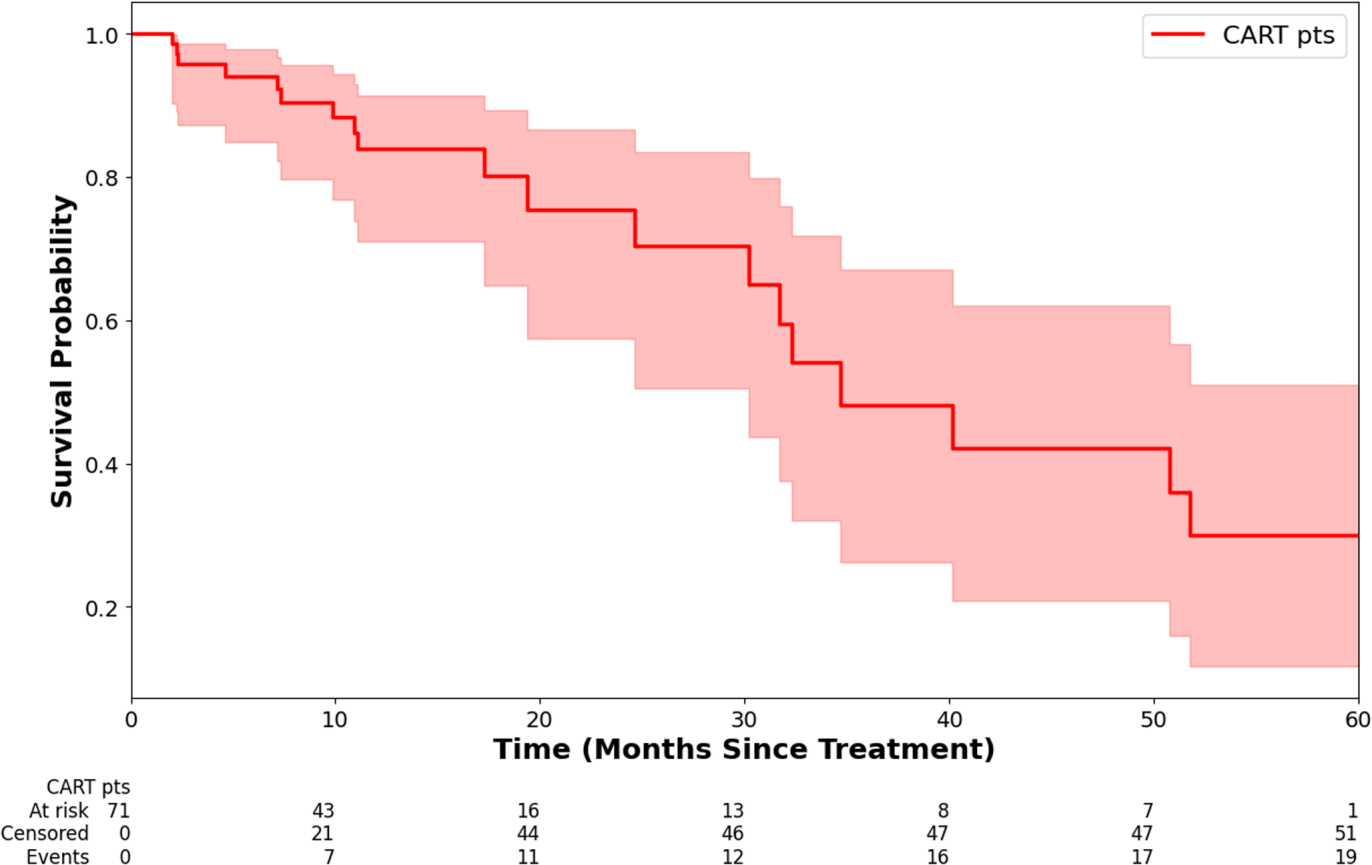
Kaplan-Meier survival curve post CAR-T treatments up to 5 years post-treatment. Shaded areas represent 95% confidence intervals. Acute mortality was excluded

Figure 1 shows the Kaplan-Meier survival curve. The cumulative mortality 5 years post-CAR-T was 34.18%. KM survival curves stratified by CR vs. non-CR, CRS vs. without CRS, and ICANS vs. without ICANS are shown in Supplemental Fig. 1. Patients with CR, CRS, and ICANS had qualitatively higher survival probability. The effects of CRS and ICANS appeared to be delayed, with bigger differences in the survival curve in later years.

Multivariate analysis was performed with covariates in Table 2 that showed group differences in the univariate analysis. Table 3A shows the odds ratios for complete remission post-CAR-T treatment. There were no variables that were significantly associated with complete remission after adjusting for confounders. Table 3B shows the adjusted ratios for mortality 3 years post-CAR-T treatment. Patients with CAR-T CR were markedly less likely to die (aHR = 0.13, 95%CI [0.04, 0.43]) than non-CR patients. Patients with pre-existing CHF and CKD were associated with a higher risk of mortality. The presence of mutation, prior ASCT, or prior > 2 chemo lines was not significantly associated with mortality risk. The univariate analysis for mortality 3-year post-CAR-T is also shown in Supplemental Table 2.

**Table 3:** (A) adjusted odds ratios from logistic regression for complete remission post CAR-T treatment. (B) multivariate adjusted hazard ratio for mortality at 3 years post CAR-T treatment

(A)	aOR (95%CI U-L)	*p*-value
CHF	0.77 (0.22–2.66)	0.676
CKD	0.51 (0.10–2.60)	0.423
Mutation	0.88 (0.31–2.48)	0.810
Prior ASCT	2.36 (0.70–7.96)	0.167
Prior > 2 Chemo Lines	1.25 (0.33–4.72)	0.743
(B)	aHR (95%CI)	*p*-value
CAR-T CR	0.13 (0.04–0.43)	0.00
CHF	5.63 (2.13–14.85)	0.00
CKD	4.13 (1.64–10.45)	0.00
Mutation	1.38 (0.50–3.80)	0.53
Prior ASCT	1.96 (0.61–6.33)	0.26
Prior > 2 Chemo Lines	0.79 (0.16–3.79)	0.77

### Blood biomarkers

We further investigated whether blood biomarkers before and during CAR-T treatments were predictive of mortality 3 years post-CAR-T therapy. Supplemental Table 3A presents the median values and interquartile ranges (IQR) for each biomarker pre (closest but up to 3 months pre-CAR-T) and during (0–31 days after CAR-T) treatment. WBC, lymphocyte count, platelet count, monocyte percentage, PT, INR, creatinine, ALT, total bilirubin, albumin, and calcium significantly differed between pre- and post-CAR-T (all *p* < 0.05). After adjusting for covariates, only high LDH pre- (aHR = 4.90 [1.77–13.65]) and during CAR-T (aHR = 5.05 [1.60–15.91.60.91]) therapy were significantly associated with 3-year mortality (Supplemental Table 3B).

### Complications

We also investigated CAR-T related complications. ICANS was significantly correlated with CRS, ICU, and sepsis occurrence. Pulmonary complication was significantly correlated with ICU and sepsis occurrence (*p* < 0.05, Table 4). Cardiac and GI complications were not significantly correlated with CRS, fever, ICU, and sepsis occurrence (*p* > 0.05).

**Table 4:** Associations between acute changes and clinical complications following CAR-T Therapy. – The algorithm fails to converge (non-significant)

	CRS	Fever	ICU	Sepsis
ICANS	11.8 (2.5–56.0), *p* = 0.001	2.1 (0.8–5.2), *p* = 0.108	22.4 (2.8–181.0), *p* = 0.004	9.8 (2.0–46.8), *p* = 0.004
Cardio	1.8 (0.2–16.2), *p* = 0.590	0.3 (0.0–1.5), *p* = 0.126	1.8 (0.3–10.6), *p* = 0.491	1.8 (0.3–10.6), *p* = 0.491
GI	2.2 (0.3–18.9), *p* = 0.485	-	0.6 (0.1–5.2), *p* = 0.636	3.0 (0.6–14.3), *p* = 0.167
Pulmonary	2.5 (0.3–21.6), *p* = 0.400	6.9 (0.8–58.5), *p* = 0.075	4.4 (1.0–18.9), *p* = 0.048	13.8 (2.9–65.1), *p* = 0.001

**Fig. 2:**
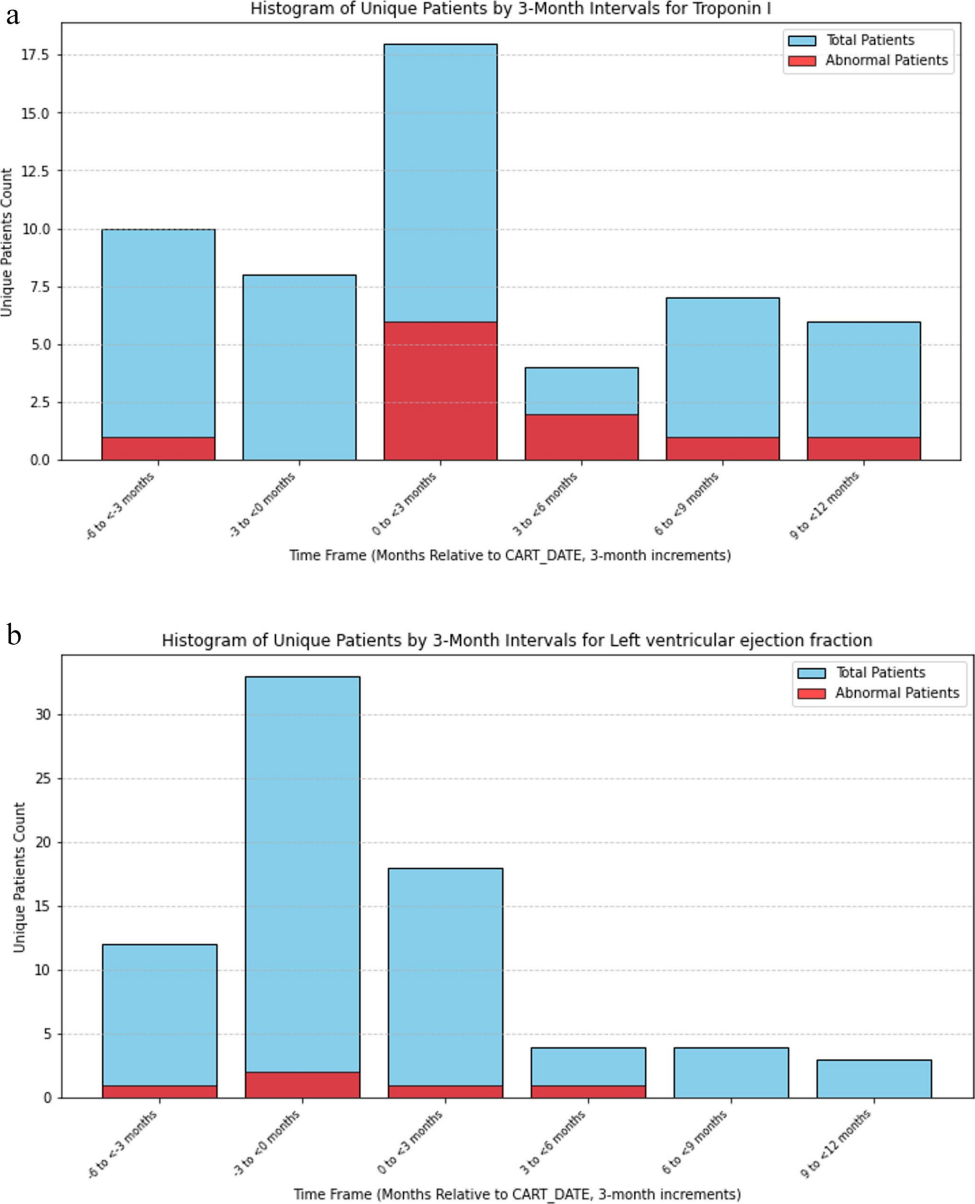
(**A**) Abnormal TNT-I and (**B**) abnormal EF at pre, during, and post CAR-T treatment

To further investigate cardiotoxicity, the percentage of patients with abnormal TNT-I at pre-, during, and post-CAR-T were examined (Fig. 2A). Most patients did not have abnormal TNT-I 6 months prior to CAR-T. Out of 71 survivors of CAR-T treatment with data, 18 patients were tested for TNT-I, of which 6 had abnormal TNT-I and were within 3 months of CAR-T treatments, and 2 had abnormal TNT-I 3–12 months post-CAR-T.

Only a few patients had prior abnormal EF (Fig. 2B). Many patients had clinically indicated EF measurements before (32/71) and after (18/71) CAR-T, suggesting cardiac function was monitored. Two had abnormal EF before CAR-T treatment. Out of 71 survivors of CAR-T treatment, 18 patients had EF measurements, of which only 1 had abnormal EF within 3 months, and 1 had abnormal EF 3–6 months after.

## Discussion

This study reported real-world outcomes of patients treated with CAR-T therapy up to 5 years post-treatment in the Montefiore Health System in the Bronx, which serves a large, diverse urban population. The major findings are: 1. CAR-T treatment showed 50.63%, 16.46%, and 22.78% complete, partial remission, and progression of disease, respectively. 2.The cumulative mortality during treatment and at 1-, 2-, and 5-year post–CAR-T treatment was 10.13%, 21.52%, 24.05%, and 34.18, respectively. 3. None of the variables investigated were significantly associated with CAR-T complete remission 4. Patients with CAR-T complete remission were markedly less likely to die (aHR = 0.13, 95%CI [0.04, 0.43]) compared to those without. Although socioeconomic status was not associated with mortality outcome (*p* >0.05), pre-existing comorbidities (CHF and CKD) significantly contributed to mortality 5. Major acute CAR-T complications included CRS (75.95%), neutropenic fever (58.23%), sepsis (18.99%), ICU treatment (18.99%), ICANS (49.37%), and cardiotoxicity (10.13%).

### Baseline patient characteristics

Prior to CAR-T, 41.77% and 40.51% of our patients showed progression of disease and relapse, respectively, 15.19% had 2 chemo lines, 34.18% had mutations, and 24.05% had prior autologous stem cell transplant. A phase II study of axicabtagene ciloleucel (axi-cel) reported that 76% of patients had baseline characteristics of disease refractory to 2 or more lines of prior therapy, and 21% of patients had relapse following autologous stem cell transplant [28]. Another study reported that prior to CAR-T, 27.3% had relapsed and 72.7% had stable or progressive diseases. 29.5% of patients infused with CAR-T had mutation, and 38% had 2 or more chemo lines [29]. Another multicenter analysis of 130 patients receiving CAR-T treatment demonstrated that about 32.8% had mutation, and 33.1% had undergone a previous autologous stem cell transplant [30].

Our cohort consisted of 24.05% of patients who were non-Hispanic black, 32.91% who were non-Hispanic white, and 29.11% who were Hispanic. Our data adds meaningful insight into CAR-T outcomes among racially and ethnically diverse patients, a population historically underrepresented in both clinical trials and real-world cohorts [31]. For example, non-Hispanic Black individuals made up only 5% of participants globally and 6.4% of U.S. participants in the ZUMA-7 trial [31, 32]. In a multicenter real-world cohort of 466 patients reported by Karmali et al., 87% were White, 7% were African American, 6% were Asian, and just 2% were Hispanic, even though nearly three-quarters were treated outside of clinical trials [31, 33]. Another large real-world analysis of 1,389 axi-cel recipients reported similarly disproportionate representation, with 81% White, 5% African American, 6% Asian, and 11% Hispanic patients [31, 34]. These patterns suggest persistent disparities in referral, access, and treatment uptake, rather than differences in disease incidence alone [31]. By reporting outcomes from a cohort that is predominantly non-White and medically complex, our study helps fill a critical gap in the CAR-T literature.

Our cohort had a comparatively higher prevalence of major comorbidities than those reported in the literature. The NIS database consisted of an obesity rate of only 6.7%, which was four times lower than our cohort [12]. Another study with a comparable sample size (*n* = 78) showed a hypertension rate of 26%, a diabetes rate of 12%, and a congestive heart failure rate of 5% [35]. Our patient cohort has nearly twice the rate of hypertension, triple the rate of diabetes, and quadruple the rate of CHF, which could have contributed to comparatively poorer outcomes.

Despite the higher burden of pre-existing conditions in our cohort, such as hypertension, CHF, and CKD, our complete remission and survival rates are broadly consistent with those reported in real-world and trial settings. This suggests that CAR-T therapy can yield durable benefits even in medically complex patients and reinforce its applicability outside of highly selected trial cohorts. This may be reassuring for clinicians managing similar patients in real-world settings.

### CAR-T treatment responses

The degree of response to CAR-T treatment is consistent with a study of 1297 patients, which found that 55.5% had complete remission and 17.5% partial remission with axi-cel therapy [36]. A meta-analysis including 984 patients across 16 studies reported 74% complete remission [37]. Despite group differences in CR observed in the univariate analysis, no variables remained significantly associated with CR after adjusting for confounders. This could be because of the small sample size and/or a relatively large number of confounds that contributed to heterogeneous outcomes. It also suggests that patient-specific and disease-related factors influencing CAR-T response are complex. The lack of strong predictors for CR underscores the need for further research to identify reliable biomarkers for response stratification.

### Mortality

Mortality during CAR-T therapy was 10.13%, consistent with a few previous studies [14, 38]. By contrast, only a few CART studies report longer-term mortality for DLBCL. One study of 275 patients treated with axi-cel showed a 5-year non-relapse mortality of 16.2% [39], about half the rate observed in our cohort. Another study of 130 patients found a 1-year lymphoma-related mortality of 28.5% [30], similar to our study. A meta-analysis included 16 studies reported 53% one-year progression-free survival [37]. Differences in findings are likely influenced by but not limited to differences in study design (clinical trials vs. real-world data), patient characteristics (including pre-existing conditions and socioeconomic factors), treatment history, cancer stage, analysis methods, and follow-up duration. We found that socioeconomic status was not associated with mortality outcomes, although it is possible that this study is underpowered to test such an association. Notably, our cohort had a comparably higher prevalence of pre-existing comorbidities, which contributed to the higher mortality observed compared to other studies mentioned above. Indeed, pre-existing CHF and CKD were associated with higher mortality risk. These results suggest that patient selection and pre-treatment optimization may play critical roles in improving outcomes, particularly for those with significant comorbidities.

Another major finding is that CAR-T complete remission is an independent predictor of reduced mortality, reinforcing the importance of achieving durable responses for long-term survival. No prior studies reported adjusted mortality risk due to CAR-T CR with which to compare. Interestingly, factors such as genetic mutations, prior ASCT, and multiple prior chemotherapy lines were not significantly associated with mortality. While prior research has suggested mixed effects of these variables, our findings indicate that treatment history alone may not be a strong predictor of survival in this cohort. Given the heterogeneity of CAR-T outcomes, future studies with large and diverse populations are needed to refine prognostic models and develop tailored treatment strategies.

### Blood biomarkers

Significant changes in WBC, lymphocyte count, platelet levels, monocytes, PT, INR, creatinine, ALT, total bilirubin, albumin, and calcium were observed between pre-treatment and during CAR-T therapy. These shifts reflect the immunologic and metabolic alterations induced by CAR-T therapy and highlight the potential of routine blood markers as accessible prognostic indicators. Among the biomarkers analyzed, only elevated LDH prior to and during CAR-T therapy was significantly associated with increased adjusted three-year mortality risk. High LDH, a marker of cellular turnover and tumor burden, has been previously linked to poor prognosis in hematologic malignancies and may indicate more aggressive disease or a heightened inflammatory state that impairs treatment efficacy [40, 41]. Although several biomarkers showed significant differences before and during CAR-T therapy, most were not independent predictors of long-term mortality. As our sample size is small, our findings on blood biomarkers need to be interpreted cautiously. Nonetheless, our findings also underscored the potential role of blood biomarkers in predicting long-term survival following CAR-T therapy.

### CAR-T treatment complications

Prevalence of CAR-T treatment complications is comparable to a multicenter analysis of 121 recipients of CAR-T therapy, in which 78.5% of patients experienced CRS, 23.1% of patients required ICU treatment, and 57.8% of patients experienced neurotoxicity [30]. In another study including 960 CAR-T recipients, neurotoxicity was reported in 30.9%, sepsis in 10.2%, and fever in 49.4% [14].

There is evidence of systemic inflammation and endothelial dysfunction contributing to ICANS, and these included evidence that blood-brain barrier disruption, mediated by elevated levels of systemic and CNS-specific cytokines such as interferon-gamma, interleukin-6, and − 8 [42], as well as elevated levels of astrocyte-specific markers in cerebrospinal fluid [43]. Neuroimaging findings associated with ICANS have also been reported [42–49]. Heterogeneous MRI abnormalities include regional white matter hyperintensity, contrast enhancement, and cytotoxic and vasogenic edema throughout the brain in some patients treated with CAR-T. Although most MRI abnormalities were reported to resolve soon after CAR-T treatment, the long-term implications of these neuroimaging findings are unknown.

In contrast to ICANS, cardiac complications were comparatively rare. We further evaluated the percentage of patients with abnormal TNT-I, a marker for acute cardiotoxicity, and found that patients with pre- and post-CAR-T abnormal TNT-I were rare. Patients with pre- and/or post-CAR-T abnormal EF were rare and likely pre-existing, suggesting that CAR-T does not cause significant short- or long-term EF dysfunction. These findings are consistent with a recent review [10].

There was a significant correlation between ICANS and CRS, ICU admission, and sepsis, aligning with prior studies suggesting that severe neurotoxicity often occurs in the setting of heightened systemic inflammation. CRS is a well-documented driver of ICANS, likely due to cytokine-mediated endothelial dysfunction and blood-brain barrier disruption [10], reinforcing the need for early intervention strategies to mitigate severe toxicity. These findings highlight the complex interplay between CAR-T-related toxicities, particularly the associations between neurological and pulmonary complications with severe systemic events.

Notably, the KM survival curves showed the effects of CRS and ICANS to be delayed, with larger differences in survival curves in later years. The delayed survival benefit in patients with CRS and ICANS supports evidence that immune activation may enhance CAR-T efficacy, despite early toxicity risks [4, 49]. While severe CRS and ICANS are linked to morbidity, controlled inflammation may contribute to prolonged survival. This underscores the need for better risk stratification and toxicity management.

Similarly, the association between pulmonary complications, ICU admission, and sepsis suggest that respiratory failure may be linked to systemic inflammatory responses rather than being an isolated event. This is consistent with prior research indicating that CAR-T patients experiencing severe infections or inflammatory syndromes are at higher risk for pulmonary complications, likely due to endothelial leakage, immune-mediated lung injury, or infection-related respiratory distress.

In contrast, the lack of significant associations for cardiac and gastrointestinal complications suggests that these toxicities may arise independently of CRS and other systemic events. This finding highlights the multifactorial nature of CAR-T-related toxicities, where some complications may be more directly tied to inflammatory responses, while pre-existing conditions, treatment history, or direct cellular toxicity may influence others. Further studies are needed to refine risk prediction models and develop targeted toxicity management strategies to improve patient outcomes.

### Limitations

This study has several limitations. Although our study is one of the largest studies using real-world data with relatively long follow-up, our sample size is small, as CAR-T therapy is only recently available.

The dataset is derived from a single academic medical center serving a predominantly urban, socioeconomically disadvantaged, and medically complex population. As such, our findings may not be generalizable to suburban, rural, or healthcare settings serving more socioeconomically advantaged populations. Similarly, our cohort’s racial and ethnic diversity may limit applicability to less diverse populations. Although we adjusted for major clinical covariates using multivariable competing risks regression, residual confounding is likely in any retrospective observational study. These results should therefore be interpreted with appropriate caution.

## Conclusions

These findings provide insight into both the promise and challenges of CAR-T therapy in a racially and ethnically diverse, medically complex population treated in a real-world setting. While approximately half of the patients achieved complete remission, the observed rates of disease progression and long-term mortality highlight the potential importance of patient selection and supportive care strategies in similar populations. The association between complete remission and reduced mortality in our cohort suggests that CAR-T therapy can achieve durable responses even among patients with significant comorbidities. Additionally, the high prevalence of acute complications such as CRS and ICANS, and their correlation with ICU admission and sepsis, points to the need for further study on early identification and management of toxicity in diverse clinical environments. Overall, these findings contribute to the growing body of evidence on CAR-T use outside of controlled clinical trials and may help inform implementation efforts in medically underserved populations.

## Supplementary Information

Below is the link to the electronic supplementary material.

Supplementary Material 1 (DOCX 137 KB)

## Data Availability

Data cannot be shared openly to protect study participant privacy; data on participants may present a risk of reidentification if shared openly. Deidentified data is available upon request from corresponding author Tim.Duong@einsteinmed.edu.

## Abbreviations

ALT: Alanine aminotransferase
ASCT: Autologous stem cell transplant
AST: Aspartate aminotransferase
BUN: Blood urea nitrogen
CHF: Congestive heart failure
CKD: Chronic kidney disease
COPD: Chronic obstructive pulmonary disease
CR: Complete remission
Cr: Creatinine
CRP: C-reactive protein
CRS: Cytokine release syndrome
DLBCL: Diffuse large B-cell lymphoma
EF: Ejection fraction
GI: Gastrointestinal
ICANS: Immune effector cell-associated neurotoxicity syndrome
ICU: Intensive care
INR: International normalized ratio
LDH: Lactate dehydrogenase
MI: Myocardial infarction
POD: Progression of disease
TNT-I: Troponin-I
