# Supplementary material for: Real-world clinical outcomes of CAR-T therapy from the Montefiore health system in the Bronx
Source: Ann Hematol. 2025 Nov 13;104(12):6359–71. doi: 10.1007/s00277-025-06727-x (PMC12764600; doi:10.1007/s00277-025-06727-x)
Supplement: Supplementary file 1 — Supplementary Material 1 (DOCX 137 KB) [file 277_2025_6727_MOESM1_ESM.docx]

**Supplementary Data**

**Real-world clinical outcomes of CAR-T therapy from the Montefiore Health System in the Bronx**

Sonya Henry^1^, Ahmed Abbasi^2^, Aiman Hafeez^1^, Aditi Vichare^1^, R. Alejandro Sica^2^, Tim Q. Duong^1^

^1^Department of Radiology, Montefiore Health System and Albert Einstein College of Medicine, Bronx, NY, USA

^2^Department of Oncology, Montefiore Health System and Albert Einstein College of Medicine, Bronx, NY, USA

**Supplemental Table 1.** Normal laboratory ranges and units used in study for dichotomization (high vs low).

| White Blood Cell Count (WBC): (4.0, 11.0), k/uL |
| --- |
| Hemoglobin: (12.0, 17.5), g/dL |
| Hematocrit: (36.0, 50.0), % |
| Lymphocytes: (1.0, 3.0), k/uL |
| Platelet Count: (150, 450), k/uL |
| Monocytes: (2.0, 10.0), % |
| Neutrophils: (2.0, 7.5), k/uL |
| Prothrombin Time (PT): (9.5, 13.5), seconds |
| Activated Partial Thromboplastin Time (aPTT): (25, 35), seconds |
| INR: (0.8, 1.2), ratio |
| Blood Urea Nitrogen (BUN): (6, 20), mg/dL |
| Creatinine: (0.6, 1.3), mg/dL |
| Aspartate Aminotransferase (AST): (10, 40), U/L |
| Lactate Dehydrogenase (LDH): (140, 280), U/L |
| Alanine Aminotransferase (ALT): (7, 56), U/L |
| Total Bilirubin: (0.1, 1.2), mg/dL |
| Albumin: (3.5, 5.2), g/dL |
| Magnesium: (1.7, 2.3), mg/dL |
| Calcium: (8.5, 10.5), mg/dL |
| Phosphate: (2.5, 4.5), mg/dL |
| Sodium: (135, 145), mEq/L |
| C-Reactive Protein (CRP): (0, 10), mg/L |

**Supplemental Table 2.** Univariate hazard ratio predicting mortality 3 years post-CART-T

| Demographics | HR (95CIs) | P value |
| --- | --- | --- |
| Age > 60 | 1.0 (0.4–2.9) | 0.953 |
| Male | 3.1 (1.0–9.8) | **0.049** |
| Black | 1.9 (0.6–5.4) | 0.258 |
| White (non-Hispanic) | 0.4 (0.1–1.4) | 0.151 |
| Hispanic | 1.1 (0.4–3.2) | 0.873 |
| Other race | 1.0(0.2-4.4) | 0.997 |
| **Treatment History / Disease Characteristics** |  |  |
| Mutation (Double hit/more) | 2.7 (1.0–7.2) | 0.052 |
| Prior >2 chemo lines | 0.84 (0.19-3.77) | 0.822 |
| Prior ASCT | 0.67 (0.19-2.37) | 0.54 |
| **Comorbidities** |  |  |
| Obesity | 2.6 (1.0–7.1) | 0.058 |
| Smoking | 0.8 (0.3–2.1) | 0.642 |
| Hypertension | 1.1 (0.4–3.2) | 0.861 |
| Diabetes2 | 2.6 (1.0–7.1) | 0.059 |
| CKD | 3.8 (1.3–11.2) | **0.016** |
| CHF | 4.2 (1.6–11.2) | **0.004** |
| Respiratory Failure | 1.7 (0.6–4.8) | 0.353 |
| COPD | 1.1 (0.4–3.4) | 0.89 |
| Asthma | 2.3 (0.7–7.3) | 0.152 |
| **Acute CAR-T Treatment Complications** |  |  |
| CRS | 0.6 (0.2–1.6) | 0.284 |
| Neutropenic Fever | 2.4 (0.8–7.4) | 0.136 |
| Neurological (ICANs) complications | 1.4 (0.5–3.7) | 0.518 |
| GI Complications | 0.3 (0.0–2.5) | 0.273 |
| Pulmonary Complications | 1.9 (0.4–8.9) | 0.393 |
| Cardiotoxicity (abnormal TNT-I) | 3.2 (1.0-10.1) | **0.047** |
| ICU | 0.4 (0.1–3.3) | 0.423 |
| Sepsis | 1.6 (0.4–7.4) | 0.537 |
| **Response to CAR-T Treatment** |  |  |
| Complete Remission | 0.3 (0.1–0.9) | **0.025** |
| Partial Remission/Response | 0.8 (0.2–3.6) | 0.773 |

**Supplemental Table 3.** (A) Laboratory values before (closest within 3 months) and during (0-30 days) CAR-T treatment. (B) Hazard ratios for mortality at 3 years post-treatment using abnormal blood biomarkers measured at baseline and during treatment. Red texts indicate a statistical difference between before and during CAR-T measurements by the Wilcoxon statistical test. ns: not significant.

**(A)**

| **Complete Blood Count & Differential** |  | Before (Up to 3 months) | During (0-31 days) |
| --- | --- | --- | --- |
|  |  | Median (IQR) | Median (IQR) |
| White Blood Cell Count (WBC) - k/uL | 78 | 4.84 (3.75-6.35) | 3.07 (2.10-4.15) |
| Hemoglobin - g/dL | 78 | 10.70 (9.27-12.04) | 9.42 (8.44-10.80) |
| Hematocrit - % | 78 | 32.42 (28.35-37.13) | 28.80 (25.68-33.20) |
| Lymphocytes - k/uL | 78 | 0.68 (0.40-0.95) | 0.28 (0.17-0.46) |
| Platelet Count - k/uL | 78 | 163.57 (126.89-208.95) | 87.38 (50.95-142.32) |
| Monocytes - % | 78 | 9.33 (7.26-12.61) | 15.02 (11.55-20.40) |
| Neutrophils - k/uL | 78 | 3.38 (2.62-4.42) | 2.10 (1.19-2.96) |
| **Coagulation Tests** |  |  |  |
| Prothrombin Time (PT) - seconds | 40 | 13.30 (12.98-14.01) | 14.16 (13.70-15.71) |
| Activated Partial Thromboplastin Time (aPTT) - sec | 40 | 29.20 (27.15-31.84) | 29.70 (27.33-32.89)ns |
| INR - ratio | 40 | 1.00 (0.98-1.05) | 1.10 (1.03-1.25) |
| **Kidney** |  |  |  |
| Blood Urea Nitrogen (BUN) - mg/dL | 78 | 13.64 (11.00-16.70) | 12.09 (9.44-17.98)ns |
| Creatinine - mg/dL | 78 | 0.83 (0.72-0.95) | 0.75 (0.66-0.90) |
| **Liver** |  |  |  |
| Aspartate Aminotransferase (AST) - U/L | 78 | 23.50 (20.60-29.88) | 24.29 (20.86-32.72)ns |
| Lactate Dehydrogenase (LDH) - U/L | 78 | 259.31 (202.63-435.24) | 206.18 (167.44-284.42) |
| Alanine Aminotransferase (ALT) - U/L | 78 | 17.46 (13.08-28.58) | 26.29 (15.36-50.22) |
| Total Bilirubin - mg/dL | 78 | 0.50 (0.41-0.69) | 0.56 (0.42-0.74) |
| Albumin - g/dL | 78 | 3.95 (3.63-4.71) | 3.55 (3.23-3.81) |
| **Electrolytes** |  |  |  |
| Magnesium - mg/dL | 78 | 1.85 (1.76-1.97) | 1.87 (1.77-1.95)ns |
| Calcium - mg/dL | 78 | 9.07 (8.68-9.32) | 8.61 (8.15-8.89) |
| Phosphate - mg/dL | 78 | 3.17 (2.94-3.52) | 3.01 (2.80-3.33) |
| Sodium - mEq/L | 78 | 138.72 (137.57-140.00) | 137.18 (135.91-139.21) |
| **Inflammatory marker** |  |  |  |
| C-Reactive Protein (CRP) - mg/L | 73 | 1.58 (0.70-5.24) | 2.89 (1.17-5.04)ns |

**(B)**

| Mortality 3 years | HR (95%L-95%U) | p | aHR (95%L-95%U) | p |
| --- | --- | --- | --- | --- |
| **Before Treatment** | | | | |
| Lactate Dehydrogenase (LDH) High | 4.90 (1.77-13.65) | 0.00 | 4.90 (1.77-13.65) | 0.00 |
| **During Treatment** | | | | |
| Lactate Dehydrogenase (LDH) High | 10.02(3.38-29.72) | 0.00 | 5.05(1.60-15.91) | 0.00 |
| Albumin Low | 7.56(2.29-24.93) | 0.00 | 1.03(0.32-3.25) | 0.95 |

***Supplemental Figure 1.*** *Kaplan–Meier survival curves comparing patients (A) with CR vs. non-CR, (B) with vs. without CRS, and (C) with vs. without ICANS.*

(A)


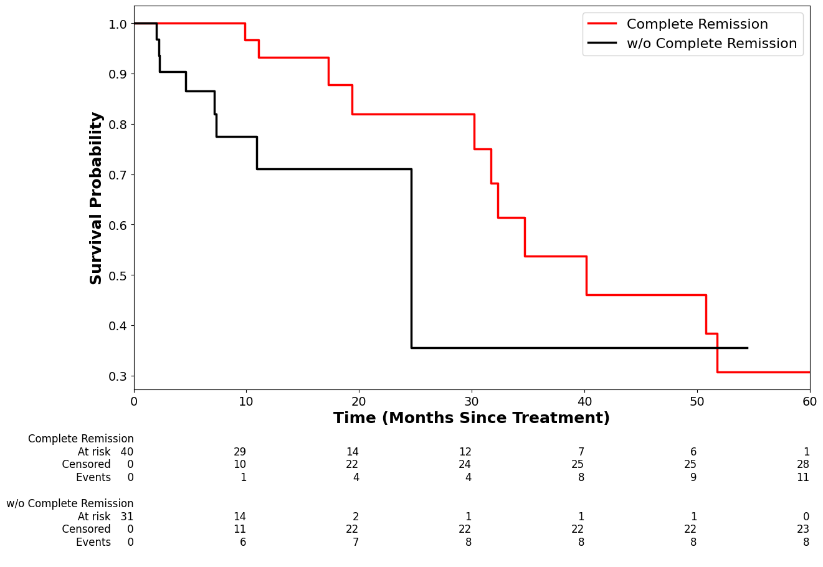


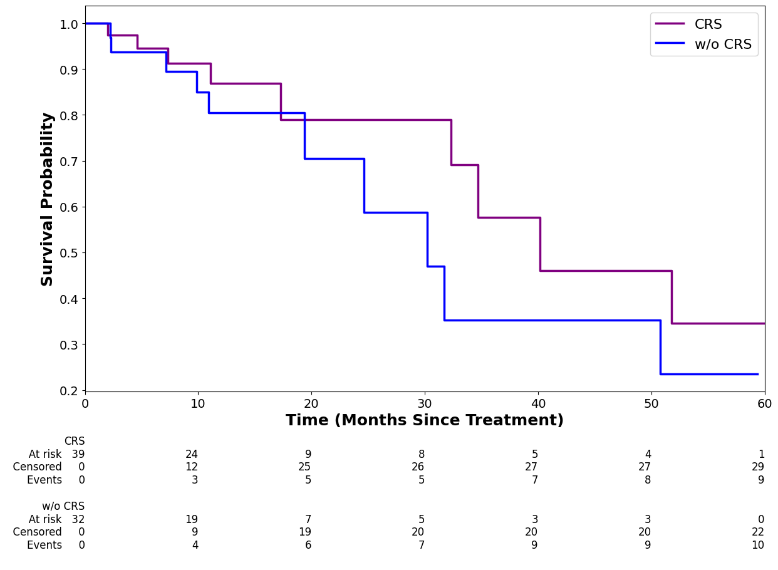
(B)


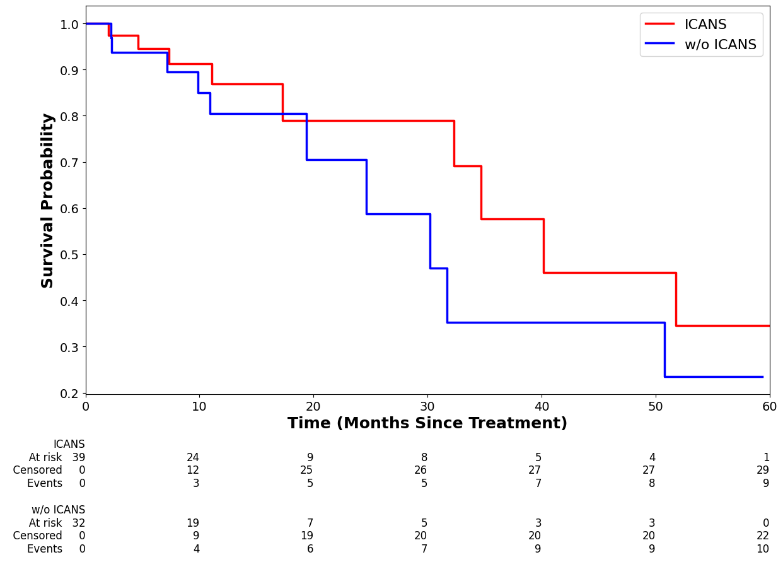
(C)
